# Observations on the stress related variations of soil radon concentration in the Gulf of Corinth, Greece

**DOI:** 10.1038/s41598-022-09441-0

**Published:** 2022-03-31

**Authors:** Vassilios K. Karastathis, George Eleftheriou, Menas Kafatos, Kanaris Tsinganos, G-Akis Tselentis, Evangelos Mouzakiotis, Dimitar Ouzounov

**Affiliations:** 1 Institute of Geodynamics, National Observatory of Athens, Athens, Greece; 2 Schmid College of Science and Technology, Chapman University, Orange, California USA; 3 Physics Department, University of Athens, Athens, Greece

**Keywords:** Natural hazards, Solid Earth sciences

## Abstract

Our observations indicate a characteristic pattern in the long-term variation of soil radon concentrations, which seems to be consistent with the expected variation of regional stress in relation to seismicity. However, it seems that the major changes in radon level begin before the rock rapture, i.e. before the earthquake occurs. These conclusions have emerged after long-term observations with continuous and thorough real-time gamma-radiation monitoring in the seismically active area of the Gulf of Corinth, Greece. The recordings acquired close to a hot spring were of very high quality, implying that the deep hydraulic flow can possibly play a key role in the pre-earthquake variation of radon level. We were able to observe outstanding examples of radon level variations before significant seismic events in the Gulf of Corinth that cannot be attributed to other external factors such as atmospheric phenomena.

## Introduction

The human attempt to forecast earthquakes through the observation of precursory phenomena began from the early historic times. Apollonius Paradoxographus (Historiae mirabiles 5), Cicero (On Divination, Book I, 50) and Diogenes Laërtius (Ι. 116) report the case of Pherecydes, the famous teacher of Pythagoras, who successfully predicted that an earthquake was to occur at three days, by examining water from a well. Besides that, Cicero mentions that Anaximander, a student of the Greek philosopher Thales of Miletus, in 550 BC, warned the inhabitants of Sparta, of an upcoming strong earthquake, and since they stayed all night outside their homes, they saw their city being completely destroyed (Cicero, On Divination, Book I, 50). Pausanias also extensively describes the existence of precursor phenomena before earthquakes when he reports the strong event of Helice in 373 BC (Pausania, Achaika, 24–7).

As huge disasters and losses in human lives often occur due to earthquakes, even in economically developed countries, human societies will persistently strive to succeed in successfully forecasting earthquakes. Although these efforts have not reached their ultimate target so far in achieving systematic, reliable and accurate short-term prediction, they are continued and intensified in recent years. Nowadays, contemporary technology is providing new means to explore and study possible earthquake precursors with new-found vigor.

The general principle and philosophy of the effort for short-term earthquake prediction is based on the consideration that such a large-scale phenomenon involves a considerable time and space of preparation depending on its magnitude^1,2^. Within this process and based on the dynamics of the plate tectonics, the potential cause for the creation and occurrence of the "precursor phenomena" is the significant change in stress, developed before the rupture^3^. Since an earthquake is proven to be related with various other physical fluctuations (such as gas emission, geodetic, electromagnetic etc.), it is also clear that it is not only limited to its "mechanical" or "dynamic" nature but also causes a number of other phenomena, several of which are well documented with numerous measurements^4–6^. A concise and thorough presentation of the earthquake precursors has been compiled by Cicerone et al.^7^ Of all, perhaps, the precursory phenomena that have been proposed, the variations in the noble radioactive gas Radon (222Rn) is what has undoubtedly been the most-discussed in the past, with extensive literature and a wealth of data. Despite the fact that first evidence came quite early, and rather unintentionally, when just after the Uzbekistan's Tashkent earthquake in 1966, Soviet scientists correlated the radon changes with the associated seismic events sequence^8^ and in spite of intensive research efforts in the decades that followed, this pre-earthquake process has not yet been exploited as an actual earthquake forecasting tool.

In related literature, there are many publications that claim to describe some connection between radon variations and upcoming earthquake events; however, such an association has been well documented only in a limited number of cases. Experimental research has shown a particularly important and direct dependence of radon changes on external factors such as variations of pressure, wind, temperature, etc^9,10^. Since atmospheric variations occur on both, a daily and a seasonal basis reaching occasionally extreme levels, monitoring of all atmospheric parameters and a long-term correlation with the soil radon concentration is necessary. The problem of assessing an anomaly and attributing it to an earthquake becomes even more difficult, if we take into consideration the fact that the choice of the occurrence time of the precursor phenomenon is quite arbitrary, without any specific rule. If, in simple terms, there is an anomaly in radon concentration, before weeks or just a few hours before an actual earthquake, scientists could possibly interpret it as a precursor of this event. It is, therefore, obvious that a long-term time series of radon concentration measurements is required, in different locations, in order to clarify the correlation between radon variation ​​and other external factors before finally interpreting correctly an actual signal. Systematic studies have been carried out in the past with extended networks in Japan, China and other countries^11^. Observations are often reported for earthquakes very distant from the epicenter, where no significant changes of stress are expected^2,12^ and also gas transfer to large distances could not be realistic due to the short half-life of radon (3.82d)^13^. Additionally, concurrent radon recordings appear to have strong local features i.e. two relatively neighboring radon stations may considerably differ in measurements^14^ as is the case with most preseismic signals that exhibit "response heterogeneity"^15^.

According to the theory of the dilatancy-diffusion model^3,16,17^, variations in the stress field and growth of cracks in rocks are proposed as the main reason of changes in radon concentration. Giardini et al.^18^ have also been led to similar findings for other gasses in the earth’s crust. Experimental data obtained under low and high level filling of the Roselend reservoir in the French Alps support this hypothesis quite well^19^. Besides, data from the Izu-Oshima-kinkai earthquake (M7.0) on January 14, 1978^11^ showed that the measured radon changes were consistent with the deformation measured by strainmeters and also with the temperature and level variations of the aquifers. Similar work has been done for radon measurements in the atmosphere in the case of the Kobe earthquake, in 1995^20–23^. Although the relation between stress and radon concentration in the reservoir experiments appears to be possible, the values of the induced stresses were small and not much larger than 1 bar/10 m of water depth. Based on this, Roeloffs^24^ proposed a nonlinear behavior of rock mass at low stresses that is mainly dominated by deformation of fluid-filled void space, including cracks and faults.

Rikitake and Hamada^2^ proposed that no precursor has been observed with crustal strain under the threshold value of 10^−9^. However, this limit is by far smaller than the respective one of the regular geodetic monitoring of precursory land deformation which is not better than 10^–7^. The extent of this zone of land deformation prior to the earthquake is expected to be very small^25^. It is clear that even if the most sensitive instruments were used, such as high-resolution strainmeters, in order to monitor a possible strain due to the aseismic fault slip near the earthquakes nucleation point, this would have to be performed within a small radius of just a few kilometers from the epicenter^24^.

From the research work of Kawada et al.^20^ it was shown that the increase in radon before strong earthquakes can be related to crustal deformation, cumulative Benioff strain and porosity change which is a critical parameter for radon migration.

The relation between stress and radon variation has been investigated also on laboratory scales^26–28^. Holub and Brady^27^ showed that a uniaxial compression on a granitic rock sample proportionally increases the exhalation of radon-222 that comes out from the pore space before any cracks are appeared and off course before the rupture of the rock sample take place. In their experiment a decrease in emanation was measured during the initial loading that was attributed to the closure of the existing cracks. The pressure that led the sample to failure increased the radon emanation up to 120% since with half pressure the radon increase reached the 50%. It is worth noting that after the failure, a level 5% has remained higher than what it was in the pre-experiment state. The experiment finally produced a characteristic curve of radon variation with respect to stress.

The question remains nevertheless, weather there is any similarity between soil radon measurements and those measured in the laboratory. Jiang and Li^29^ also worked on granite samples under uniaxial stress and found that the radon emanation began to increase before failure. Furthermore a major increase in radon emanation occurred during the rupture. However, their experiments with limestone and basalt samples with low uranium (U_3_O_8_) and thorium (ThO_2_) concentrations under similar loading conditions didn’t produce similar results. King and Luo^28^ showed a possible correlation between radon emanation and stress by conducting experiments on concrete, which is very porous in comparison to granites and could better resemble materials encountered in tectonic structures and faults and suggested that the increase can be mostly attributed to the creation and development of axial dilatant microcracks which tend to increase the surface area for gas emanation and permeability. The development of axial dilatant microcracks has been proposed as pre-earthquake process by Thomas^30^.

In this article we present examples of clear changes in soil radon concentration before earthquakes. We further investigate whether these recorded anomalies follow a specific pattern, which may be related to changes in stress. We also examine whether these anomalies are recorded simultaneously with similar characteristics in neighboring stations. Our ultimate goal is to test the validity of a possible direct connection of the radon anomaly with stress changes.

**Figure 1:**
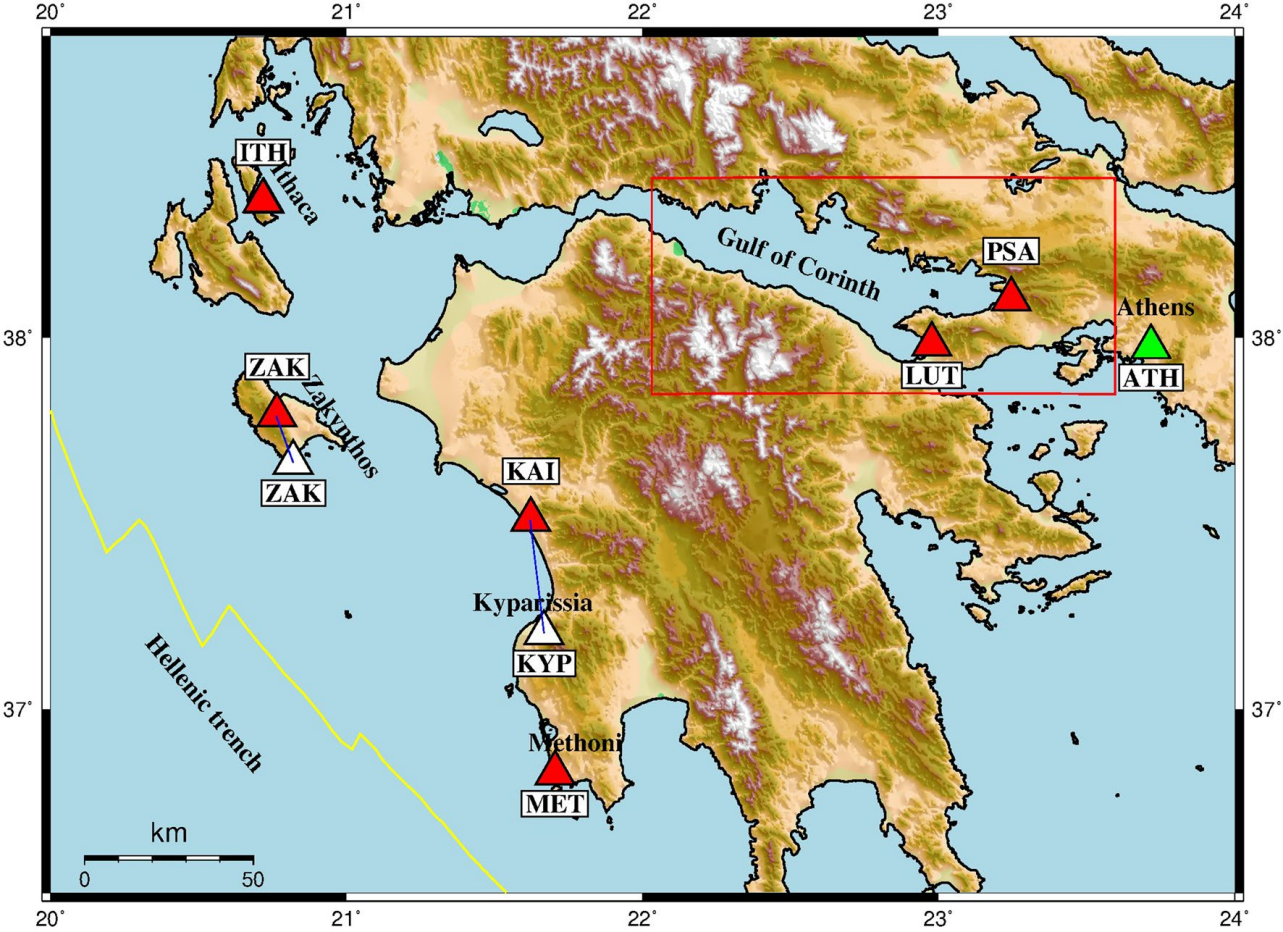
The gamma-ray station locations of the real-time network of NOA for soil radon concentration measurements (red triangles). Older station locations are also shown (white triangles). The alpha-particle station for Rn monitoring in the air is shown with green triangle. The region in the red rectangle includes the investigation area. The figure has been created by the “Generic Mapping Tools” GMT5.0 software (https://www.generic-mapping-tools.org/).

In order to monitor the variation of soil radon concentration before strong earthquakes, we installed 6 recording stations at high seismicity areas such as the eastern part of the Gulf of Corinth and also at the Hellenic trench in Western Greece (Fig. 1). The active tectonic structures of the Greek region constitute an ideal natural laboratory for the research of earthquake precursors. The investigation of the physical processes that determine the occurrence of such phenomena contributes crucially to a deep understanding of the geodynamic regime of these active regions. This understanding continuously improves the practices for a proper seismic hazard assessment of areas in direct proximity to big urban centers and highly important energy and industry infrastructures. The Gulf of Corinth and its broader area has much suffered in the past by many catastrophic and lethal earthquakes. Knowledge of the precursor phenomena can contribute decisively to earthquake forecasting.

The installation of these stations began in May 2016 and in all sites a real-time monitoring has been established. In the areas selected, significant fault zones with dense faulting have been identified by geophysical surveys^31,32^. The first stations of the network were installed in the areas of Methoni (MET), Kyparissia (KYP) (it was recently moved 25 km to the north at a neighboring geothermal site –KAI) and Zakynthos island (ZAK). The network was later extended with two other stations, in Loutraki (LUT) and Psatha (PSA), in the region of the Eastern Gulf of Corinth. This area is of high seismic potential with a series of three major earthquakes of magnitudes M6.7, M6.4 and M6.3 occurred at 2/24/1981, 2/25/1981 and 3/4/1981. The region has major faults and has not been very active in recent years. Since 2019 a new Radon monitoring station has been established in Ithaca (ITH). The radon network was mainly based on NaI(Tl) scintillation detectors for gamma spectrometry. An alpha-particle station has been also installed in Athens for measuring the radon concentration in the air of a small tunnel (ATH). More information about the instruments is given in the chapter of Methodology.

Our results showed that the most reliable recordings were obtained in the Gulf of Corinth and therefore we will mostly focus on these data by interpreting them in relation to the characteristic tectonic regime of the region.

### Tectonic setting and seismicity of the Gulf of Corinth

The Gulf of Corinth, one of Earth's most active continental rift systems is a high seismic hazard area with many catastrophic earthquakes in its history; however, its seismicity rate is not as high compared to other active tectonic systems such as the Hellenic Arc region. The long quiescent periods between the major seismic events facilitate the correlation of the possible pre-earthquake radon variations with the seismic events. The gulf is a rapidly extending region with high rates up to 1.5 cm/yr at the western part and 0.5 at the eastern one. The main tectonic structures of the Gulf of Corinth work under an extensional field and consist of regular, listric faults with an approximately E-W direction and a slope to the inner part of the gulf. In the eastern Gulf of Corinth there are also important active tectonic structures with a NE-SW direction^33^, which have been associated with strong earthquakes such as the ones on Feb 24, 1981 of Mw6.6 magnitude, Feb 25 of Mw6.3 and Mar 4, 1981 of Mw6.2 (magnitudes from Harvard CMT^34^).

**Figure 2:**
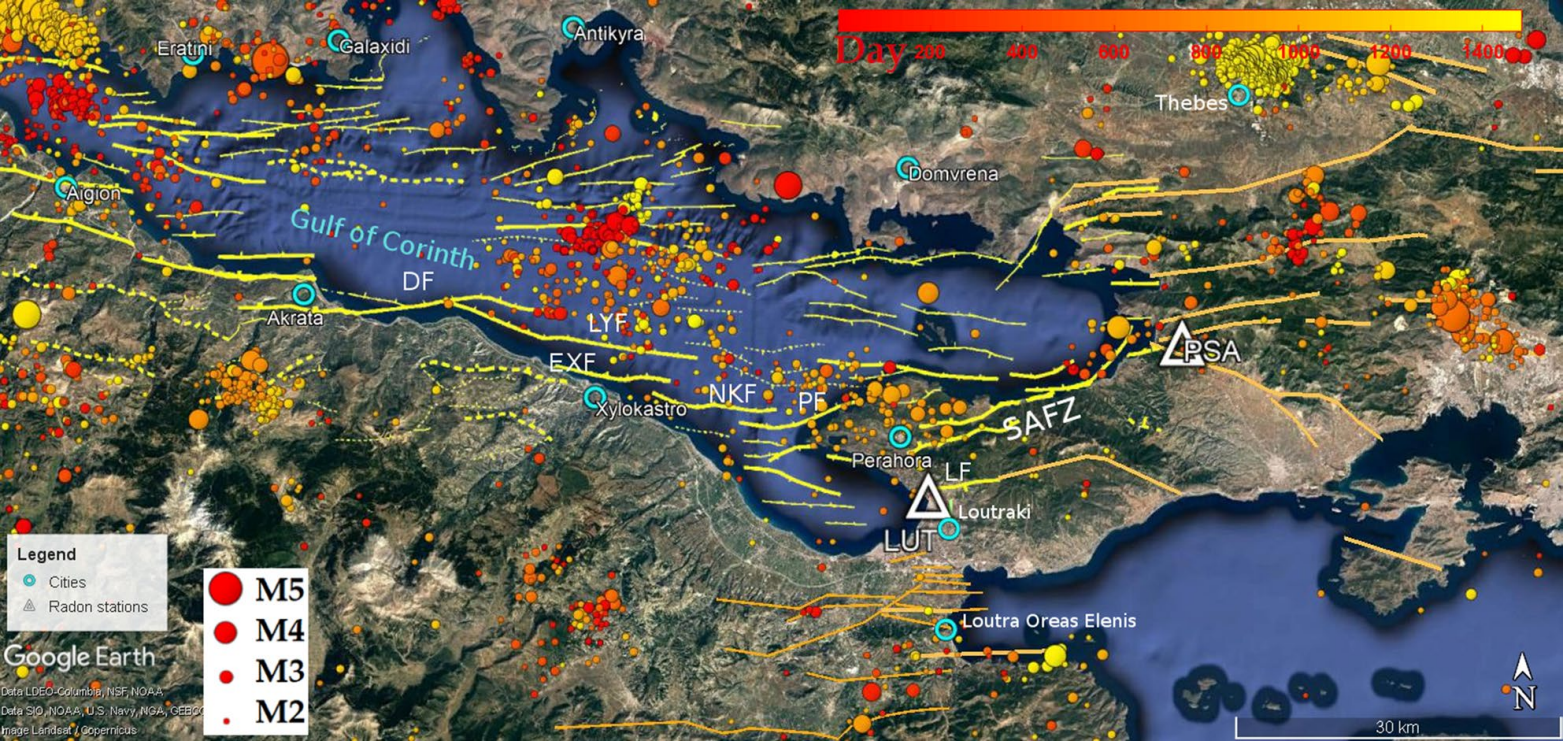
Fault zones of Gulf of Corinth as mapped by Nixon et al.^32^ (yellow). The faults of eastern and southern area have mapped as mapped by Lekkas^35^ and Ghisetti and Vezzani^36^. The north dipping North Kiato (NKF) and Perachora Faults (PF), ~ 12 km in length, form a dextral en echelon fault array with the East Xylokastro Fault (EXF) which is probably linked at depth. The South Alkyonides Fault Zone is indicated in the map as SAFZ. The seismicity map has been based on the catalogs of the National Observatory of Athens (NOA). The color indicate the time (day after Oct. 1, 2017).

Nixon et al.^32^ stressed that along the southern margin of the Gulf of Corinth basin, the north dipping North Kiato and Perachora Faults, ~ 12 km in length, form a dextral en echelon fault array with the East Xylokastro Fault which is probably linked at depth (Fig. 2 shows the faults of the region^32,35,36^). The multichannel seismic and bathymetry survey of the central and eastern Gulf of Corinth previously conducted by Taylor et al.^37^ (2011) investigated the structure and showed that the north-dipping faults of the southern basin margin are biplanar to listric with angles about 35° in the centre and 45–48° in the east near the surface. At greater depths their dip decreases to 15–20° in the centre and 19–30° in the east respectively. Although, in the past, there was a debate on the rift mechanism^38–40^, it is now mostly acceptable that the rift is asymmetric with the faults of the southern border to be mainly responsible for the grater events such as the Ms5.9 Galaxidi earthquake on November, 18 1992^41^. However, at the most eastern area of the gulf, there are also some important active antithetic faults such as the Kaparelli fault that has been associated with the Mw6.3 earthquake of Mar 5, 1981^42^ and south-dipping Loutraki fault that extends offshore westward^43^ (Fig. 2).

## Results

During the 5-year operation of the radon network, various soil radon concentration anomalies have been recorded, which in no case can all be attributed to seismic events. Station LUT (Fig. 2) has been installed since October 2017 in a shallow 2-m borehole at a site close to thermal baths’ water supply at a high proximity to hot springs. The station is located on the hanging wall of the fault that facilitates the hydrothermal flow of the thermal springs. The fault has an EW direction but extends into the sea with a NW–SE direction. Station PSA is located in the area of Psatha, in the immediate vicinity of a normal fault that is part of the great active tectonic zone of the Southern Alkyonides^33^, which is associated with the strong earthquakes of Feb 24, 1981 with Mw6.6 and Feb 25 with Mw6.3 (Fig. 2).

**Figure 3:**
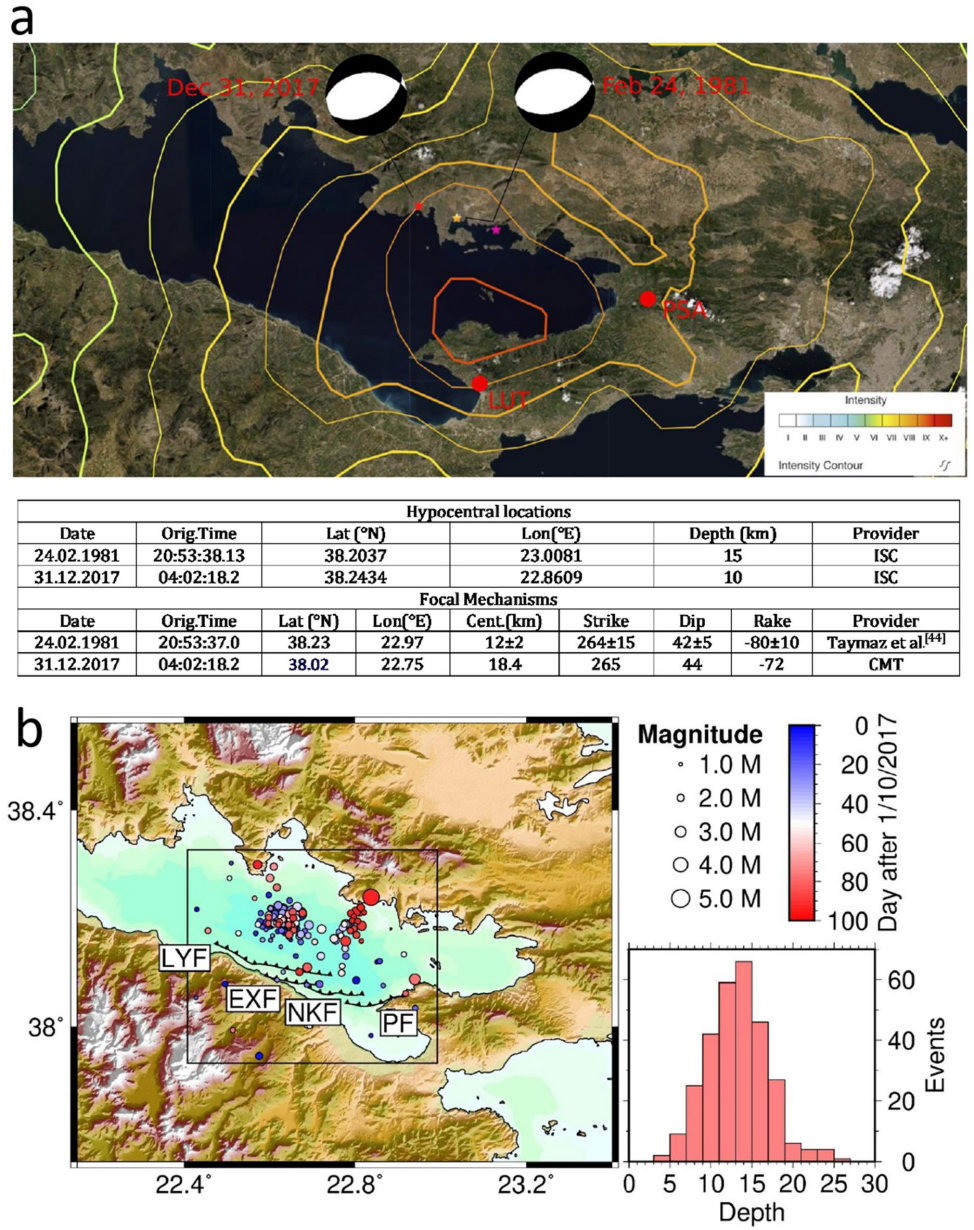
(**a**) The epicenter of Mw4.9 earthquake on Dec.31, 2017 (red star—ISC) was almost at the same place with the Mw6.6 catastrophic event of Feb. 24, 1981 (yellow star—USGS and purple star-ISC solution). The focal mechanisms were also absolutely similar. The two solutions are shown in the embedded table below. Both solutions are compatible with the same large faults that exist at south coast of the gulf. The image also depicts the Feb 24, 1981 earthquake intensity based on the Modified Mercalli Intensity Scale as calculated by USGS^45^. LUT station is in a very close proximity to the fault that is probably associated with the two events. (**b**) The seismic activity for the period between Oct. 1–Dec. 31, 2017, before the strong Mw4.9 event of Dec. 31, 2017, can be related with the activation of the low angle faults of the southern coast of the Gulf of Corinth.

Οn Dec. 31, 2017, an earthquake of a moderate magnitude Mw4.9 occurred at the northern margin of the Gulf of Corinth, just opposite to the Perachora peninsula at the same place of the Mw6.6 event of Feb 24, 1981. This seismic event was the only one with a magnitude greater than M4.5, in the area of Eastern Gulf of Corinth from 2012 until today. The earthquake caused concern among the local population because it occurred just at a distance of 13 km west of the epicenter of the Mw6.6 devastating and lethal earthquake on Feb. 24, 81, as calculated by the ISC or less than 7 km west from the corresponding one calculated by USGS (Fig. 3a). The seismic intensity curves of the Mw6.6 (Ms6.7) earthquake are also shown in Fig. 3a, where it is clear that the damage from the earthquake was mostly south, above the hanging wall of the major faults of South Alkyonides fault zone (Fig. 2).

The focal mechanism of the earthquake of 31.12.2017 as calculated by the Harvard CMT project (Fig. 3b), considers a seismic source with very similar characteristics to those of the earthquake of 24.02.1981^44^. Both the proximity of the epicenters and the similarity of the focal mechanisms support the assumption that the earthquake of 31.12.17 originated from the faults on the south margin of the gulf and probably the same fault that was activated in 1981. The event of 31.12.2017 had a foreshock activity that became quite high during October (Fig. 2). A detailed seismicity map for this period is depicted in Fig. 3b. From the figure it is clear that the events of the eastern seismicity cluster in the period just before the earthquake better fit with a NE-SW fault such as the Perachora fault that is parallel. The depths and the event locations are compatible with the expected characteristics of the PF fault. The seismicity at the western cluster that occurred between Oct.1, 2017 and Dec. 11 2017 is mostly compatible with the North Kiato and Xylokastro faults having also some events that could be attributed to the PF. The LF could also be related although it is too close to the epicenters, if we take into account the hypocentral depths shown in the histogram and the low angle dip of the faults. Therefore, we believe that there was mainly an activation of the northern dipping Xilokastro, North Kiato and Perachora faults. According to Nixon et al.^32^ these faults form a dextral en echelon fault array that is probably linked at depth. In addition, the Loutraki fault and its offshore extension practically osculate with this structure forming a narrow tectonic horst. The carbonate bedrock characterized by intense fracturing and karstic aquifer forming^46^ allows the deep fluid circulation between the two faults. Therefore, the area of LUT station seems to be directly and physically connected through the faults with the focal area of the Dec.31, 2017 event. Deep fluid circulation through the faults in the region has been identified at several sites^40,47,48^.

**Figure 4:**
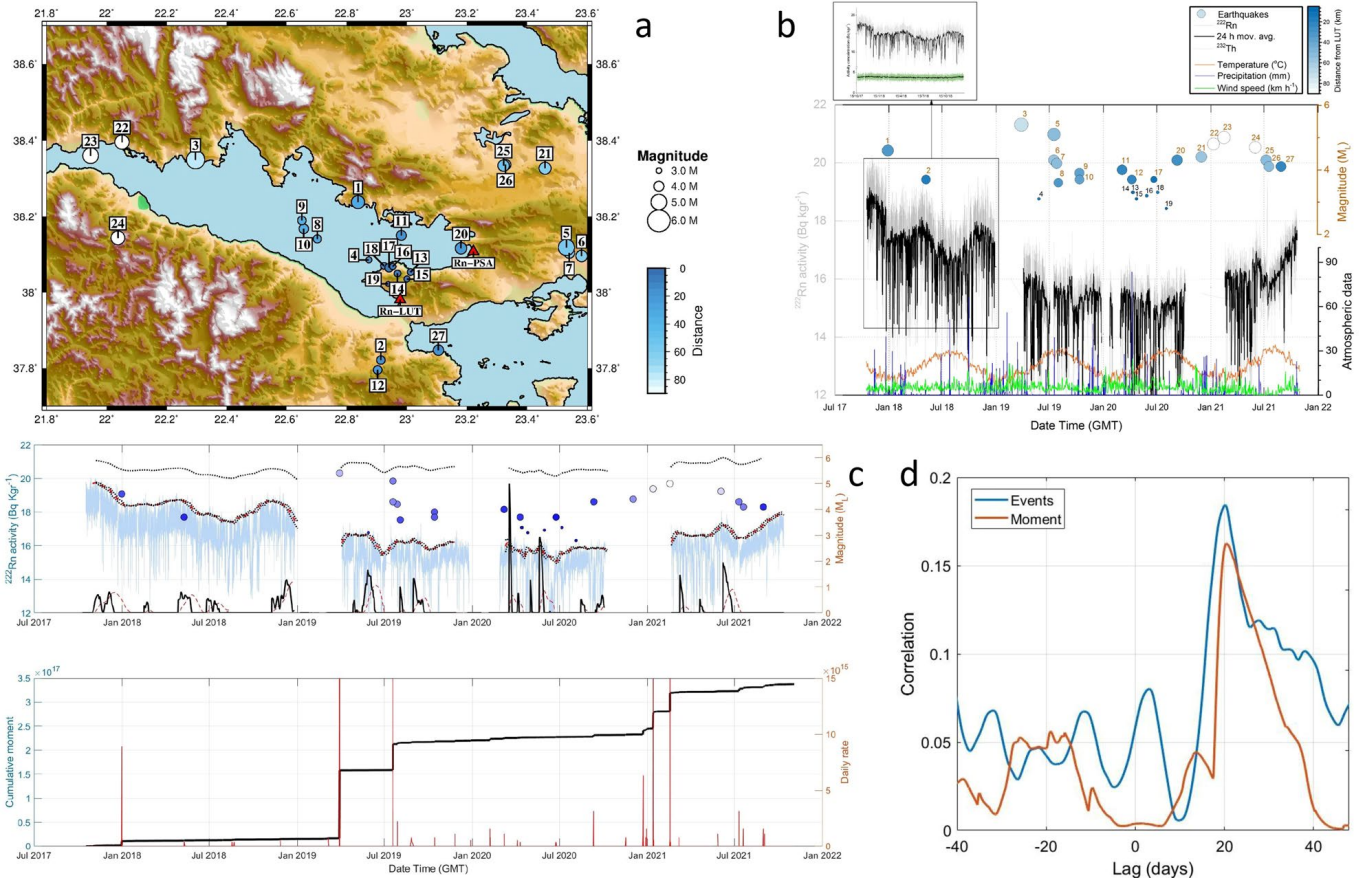
(**a**) Seismic events selected under the Dobrovolski criterion. (**b**) Radon soil concentration measurements of Loutraki (LUT) station. The general trend resembles the stress variations of the region. The abrupt drops correspond to strong earthquakes. The radon measurements are not affected by the temperature variation. The stability of thorium measurements (embedded picture) is an indirect indicator of the validity of the full spectrum analysis applied on the radon measurements. (**c**) Top panel: the identification of the radon anomalies was based on an STA/LTA algorithm applied to the envelope of the radon measurement time series. The points where after a systematic built-up of a radon increase, a rapid drop of radon level is observed, are detected by the negative values of the first derivative (red line) of the STA/LTA values. The second derivative (black line) of these indicates exactly the initiation time of the drop anomalies. Bottom panel: the cumulative seismic moment and daily rate are shown with black and red lines respectively (**d**) The result of the cross-correlation between the second derivative of radon anomalies and the times of selected events (blue line) as well as the peaks of the daily seismic moment rate.

**Figure 5:**
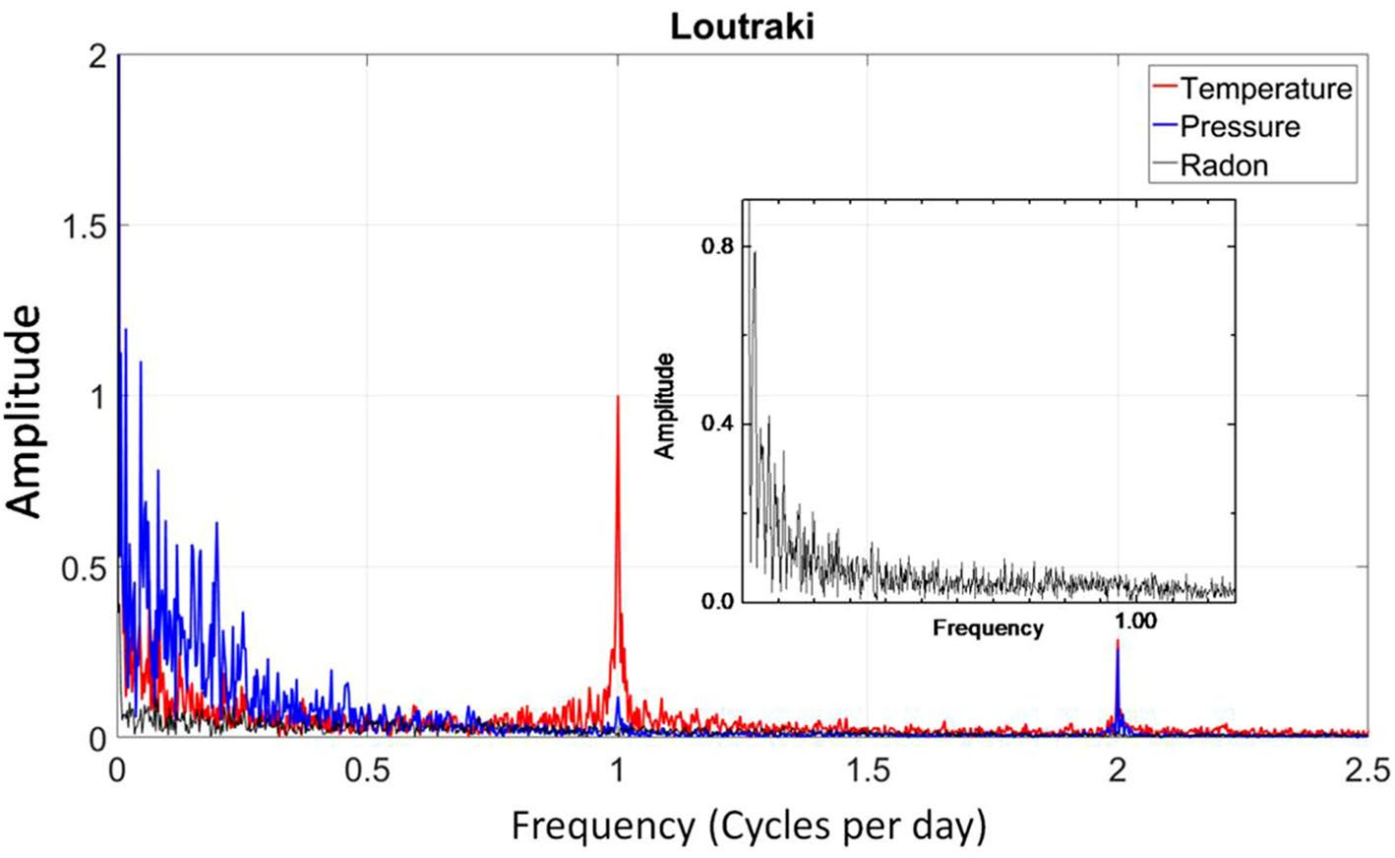
In the FFT diagram the Radon showed by a black line doesn’t follow neither the diurnal variation of temperature nor the semidiurnal one of Pressure.

The radon measurements in the LUT station are not influenced by fluctuations in atmospheric parameters such as temperature, humidity and pressure (Figs. 4, 5). This could be related with the thermal properties of the ground of the LUT site and possibly the continuous supply of heat at a constant temperature from the internal hydrothermal flow. The only exception was the effect of the winds, which sharply lowered the measurement values but only for hours, while at longer intervals the radon level was not affected. The Pearson and Spearman correlation coefficients that were computed for the LUT data between the 222Rn variation and the seasonal cycle of air temperature (Fig. 4) were both very small, at about − 0.12 and did not imply any statistically significant correlation. Similarly, small values were calculated for humidity (Pearson 0.25, Spearman 0.24).

As it can be seen in Fig. 4, in the first recording period of LUT after Oct.15 2017, the radon level appeared to be stable with a small variation between 18–20  Bq/kg with a constant average level at about 19 Bq/kg. This level remained stable until Dec. 23, 2017, when it dropped down sharply and the values then ranged between 16–18 Bq/kg. The values, however, of thorium (232Th) and potassium (40K) remained at an absolutely constant level showing the stability and reliability of the measurements (see also the chapter of Methodology). Within a week, the earthquake of Dec. 31, 2017 occurred at the coastal area opposite the Perachora peninsula. During the months following the earthquake, it was made clear that a new radon level was established. It is really important that the source was possibly the same as that of the 1 981. In this way so, we can realize how feasible and important such an observational fact is, in order to predict a similar catastrophic earthquake in the future.

**Figure 6:**
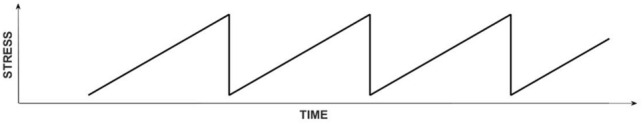
Schematic presentation of the earthquake generation process. Tectonic stress is slowly accumulated along the fault until it reaches local strength limit that is the critical stress required for failure. Then the earthquake occurs with a sharp stress drop. A new earthquake cycle follows.

After this earthquake, a gradual increase of the radon level started and continued until the end of April, 2018, when after a new event of M3.7 occurred on May 12, 2018, only 18 km far from LUT, the radon concentration dropped again in the previous lower level. After Aug. 25, 2018, the radon level started increasing again up to almost the high level that was recorded before the last event. This high radon level remained up to the end of the year 2018, when the first period of recording had been scheduled to be completed. The recording restarted on Apr. 10, 2019, after a strong event occurred in the Gulf of Corinth of Mw5.3 (CMT) Mb5.6 (ISC) magnitude on Mar. 30, 2019. The new level of the soil radon concentration was significantly lower in a similar way to the case of the Dec. 31, 2017 event. However, here it cannot be identified if the drop of the radon level occurred before the event. The second recording period had exactly the same characteristics as the first, and here the charging periods have a gradual and smooth increase in soil radon concentration and after the peak we have a strong seismic event within the zone. This sequence of increases and drops follows the pattern of the stress circle^49^. The variation of radon in fact seems to follow the stress cycle depicted in Fig. 6. However, it seems that the drop in the radon level starts with an early stage of fracture before the main shock. This pattern can be clearly observed only in the case of individual events. In case of multiple events, it is difficult to attribute any specific anomaly.

**Figure 7:**
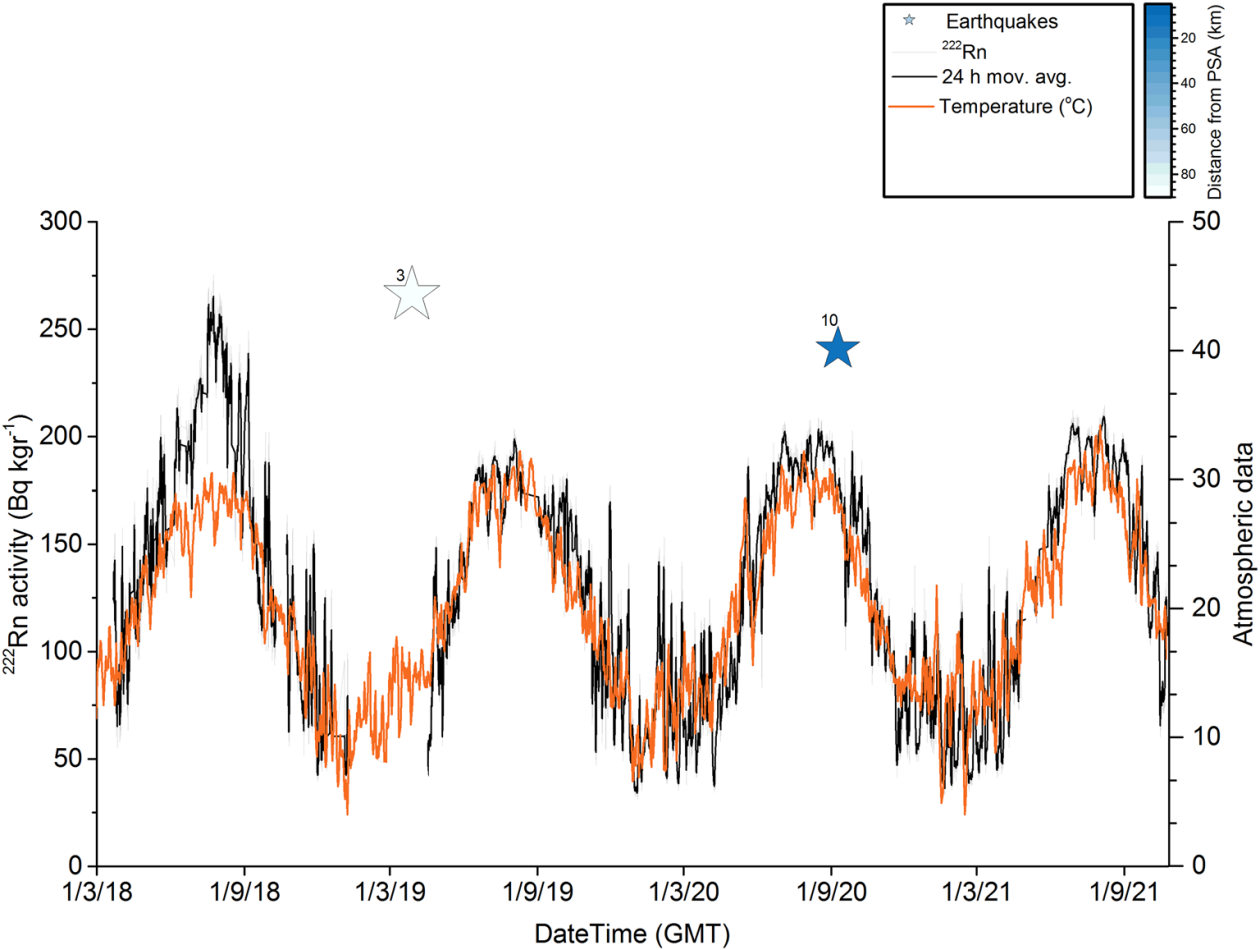
The level of the soil radon concentration at the Psatha station systematically follows the atmospheric temperature. From the overall picture of the monitoring, a substantial drop on the radon lever can be observed in the second period of the recording, after the Mw5.6 event of Mar. 30, 2019 (event No.3 in Fig. 4a). The identification of the radon anomalies is very difficult even for the closest events (event No.10 in Fig. 4a).

**Figure 8:**
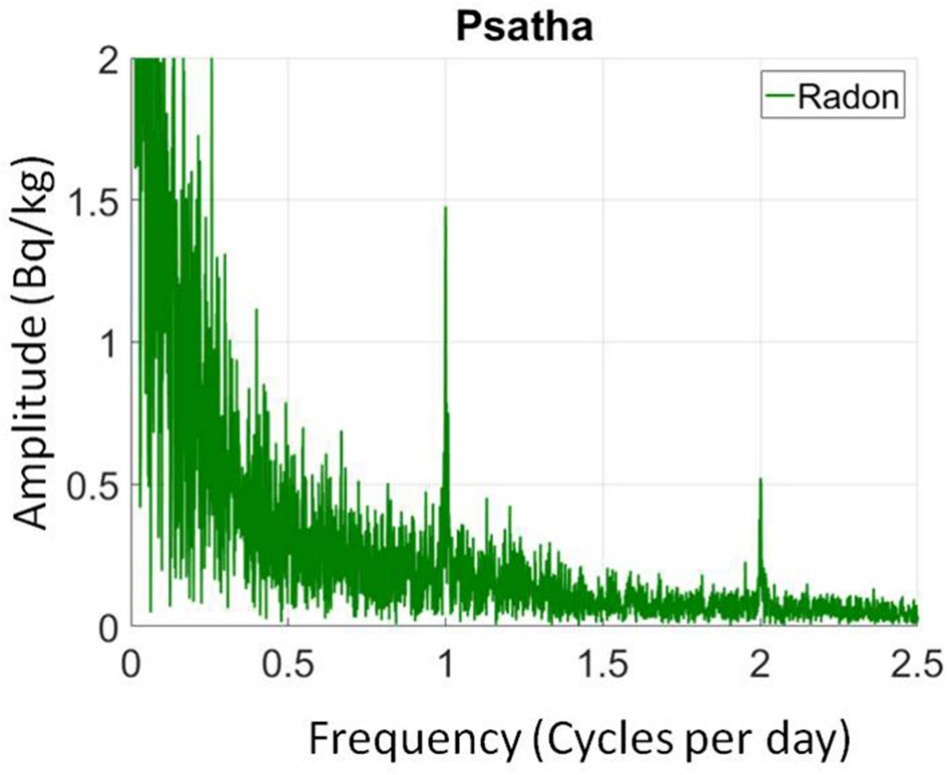
The strong correlation between temperature and the measured soil radon concentration is also obvious in the FFT spectrum from the prominent diurnal and semi-diurnal variation (peaks on 1 and 2 cycles per day).

In order to ascertain the confidence limits of the radon variations, the statistical significance of the anomalies and whether a statistically strong correlation between radon anomalies and earthquakes is obtained, we proceeded to further analysis of the data, the details of which are described in detail in the chapter of the methodology. The results are presented in Fig. 4c. The abrupt drops after a gradual increase in radon concentration were identified by an automatic algorithm using the STA/LTA function applied on the upper part of the envelop of the radon measurements and its 1st and 2nd order derivatives (see “Methodology”). The selected earthquakes according to the preparation zone criteria, the cumulative moment of the entire seismicity of the region and its daily rate are also shown in the same figure. Since the cumulative moment curve is independent of the epicentral distances of the events from the radon station, this information, which is adequate for the proper interpretation of the results, has been included with a color scale on the event symbols. The standard deviation of the values of the smoothed envelope is up to 0.20 Bq/kg and is also shown in the figure. This procedure successfully identified all the anomalies that could be visually inspected. The 2nd derivative (red line) indicates the initiation points of the anomalies and these are very well matched with the events. The anomalies of the cumulative moment and its daily rate fit well with the selected events. It should be noted that some small adjacent to the station events have strong anomalies on the radon curve but small contribution to the cumulative moment curve. In contrast some distant strong events shown clearly in the seismic moment curve do not analogously affect the radon measurements. The cross-correlation performed between the second derivative of STA/LTA (initiation of a radon anomaly) and the times of the events and also the daily moment release both showed a preferable time lag of about 20 days. Distant strong earthquakes such as the event of Mw6.7 magnitude in Zakynthos, on Oct. 25 2018, did not cause any noticeable change despite the fact that its epicenter was 230 km away, distance that could justify such a change according to studies of preparation zone determination^1^. However, a long-term monitoring with multiple strong earthquakes occurring at certain distances from the Gulf Corinth is needed to ascertain that we have a closed monitoring system related only with the tectonic zones of the Gulf of Corinth and their peripheral zones. At a short distance to the recording station LUT and at the South Alkyonides fault zone, the PSA station operates with an identical sensor. Its operation started a few months after the earthquake of Dec. 31, 2017 and a comparison between the recordings of the two stations for this event is unfortunately not feasible. However, a comparison can be made for the following time period. It should be noted here that for the specific station, the radon level is mainly affected by atmospheric seasonal changes rather than geodynamic phenomena (Figs. 7 and 8). In fact, the radon level follows the atmospheric temperature variations in such a dominant way that if we subtract this effect, we normally reach the natural reference level of radon. The Pearson and Spearman correlation coefficients for the PSA data between the 222Rn variation and the seasonal cycle of air temperature (Fig. 4) were very high, both at about 0.88 and indicate a very statistically significant correlation. Similarly, humidity showed rather high negative values (Pearson − 0.47, Spearman − 0.49).

PSA similarly to LUT completed the first recording period at the end of 2018 and it was restarted on Apr. 24, 2019. We observed that the radon level in PSA is significantly higher during the recording period of 2018 in comparison with the second part of recording. This drop in radon level was noticed also in the recordings of LUT and may be related to the event of Mar. 30, 2019.

## Interpretation and discussion of the results

Given that the difference between the two stations, LUT and PSA is only the existence of the hydrothermal flow in the LUT station, it is obviously critical to introduce the parameter of the deep hydrogeology in the whole concept. The change in deep hydrothermal flows due to the change in pressure may eventually be related with the radon change. Also stresses on confined aquifers can be easily transferred. Beside this, the inhomogeneity of the stress distribution on an aquifer forms a pressure gradient that can cause radon to move. Induced small earthquakes can also be triggered as a result of lubrication of neighboring peripheral faults by changes in deep hydrogeological flows. The question is whether the observed change in radon resulted from the change in hydrogeological fluxes within the faults or by the changes in "porosity" in the sense described by Kawada et al.^20^ caused by the stress change. It should be mentioned here that significant foreshock activity has been noticed before the strong anomaly of the event of Dec. 31, 2017 (see Fig. 2).

The hypothesis that fluid movements are associated with earthquakes and that there are some "sensitive wells" to earthquake related changes, has been introduced in the past by Wakita^50^. He even found that the shape of the signals was probably related to stress accumulation in the region. Our measurements lead us in the same direction and confirm Wakita, but they take us one step further in terms of the model that regulates the procedures.

Tsang and Niemi^51^ stressed that deep drilling confirmed that above the brittle-ductile transition, hydraulic flow may by facilitated mainly through fracture zones and clusters of varying permeability, which have been formed through tectonic processes. Moreno and Neretnieks^52^ described these deep flow paths as channel network. The seismic and rock deformation processes may change local porosity, especially near choke points between hydraulic compartments^53,54^, and this would in turn change the permeability and flow structures. A major change of the stress field can cause large flows between the hydraulic compartments, which may also justify the activation of several faults with microseismicity and also cause stress variations and radon release in relatively quite long distances justifying simultaneously locality of anomalies. That is, at relatively neighboring stations it will be possible to justify the difference in the appearance or not of a precursor anomaly in the soil radon concentration or even the existence of opposite anomalies. In fact, if the predominant factor for the occurrence of an anomaly is the hydraulic changes through the flow networks and faults as a result of a change in the stress field, it will be possible to identify such anomalies in areas connected by faulting, especially if this has a proven hydrothermal flow. It should be stressed that the faults of region of eastern Gulf of Corinth facilitate hydrothermal flow at areas of Loutraki and Loutra Oraias Elenis (Fig. 2).

A very important observation is the early drop of radon level before the earthquake rupture while in contrast to the experiments reported by Holub and Brady^27^ and Jiang and Li^29^ there was a sharp increase in radon before the rupture, which is attributed by the authors in microcracking. However, it is worth to note that in laboratory experiments, the stress was compressive, while in the cases mentioned in the Gulf of Corinth, it was purely tensile. The anomalies were almost reversed compared with the results of laboratory measurements. Even during the initial loading in the experiment of Holub and Brady^27^ a decrease in emanation was measured and it was attributed to the closure of the existing cracks. However, when the field of stress is extensional, the stresses initially tend to dilate part of the crust, increasing porosity and cracks in the fractured tectonic zone and thus facilitating the radon flow to the surface.

In the case of the Gulf of Corinth, when the early activation of the rupture begins with microcracking, the radon content also starts flowing laterally in the activation zone until it reaches equilibrium and therefore a sudden drop is expected. This drop stops with the earthquake occurrence. The radon concentration is then at new level lower than the one before the event. At this point, due to the new cycle of accumulation of tensile stresses, radon level will gradually begin to increase until the next earthquake. Smaller events occurring in the zone, or in close proximity, may release some of the accumulated stress and so, radon may fluctuate.

Combining the previous knowledge presented above, and confirming the hypothesis of Wakita^50^, we can propose a model where the tectonic stresses applied to the rock formations during the earthquake preparation phase, probably strongly affects the flow paths of a deep hydrogeological channel network system that is connected to the surface through faults and hot springs. It is possible that these changes that are caused by the water movements, facilitate at the same time the foreshock activity and also the variations of the radon outflow in neighboring areas. Such a deep hydrogeological network can transfer over long distances the information of the stress change and justify the selectivity of the sites (also stressed by Wakita^50^) where the changes in the radon concentration function as a pre-earthquake phenomenon. The stresses, apart from the direct influence they have on the porosity of the earth's crust, also change the water content in the faults. In such a model the radon variations due to earthquake preparation phase are limited in the inter-connected fault zones.

It is worth noting that during the recording period, no anomalies in the radon concentration were simultaneously detected in several stations so that they can be objectively attributed to distant strong earthquakes such as the event of Zakynthos of Mw6.7 magnitude on 25/10/2018 240 km SW far from LUT, or the Mw6.9 event of Samos on 30/10/2020, about 340 km E far from LUT, or even the double event in Elassona (M6.3, M6.0) on Mar 3 and 4, 2021 about 210 km N from LUT. Although there were two stations at relatively close distance to the Zakynthos epicenter, a very strong Mediterranean tropical-like cyclone occurred in Sep 28-Oct 3, 2018^55^ strongly affected the validity of the recordings and finally temporally destroyed the one of these.

## Conclusions

An important observation that can be deduced from the cases we examined is that the radon variation observed along with seismicity follow a pattern similar to the stress accumulation cycle.

The form of the anomalies has features in common with several other anomalies reported in the literature such as this of the earthquake of Tashkent in 1966^8^. In the case of the extensional field of the Gulf of Corinth, a gradual increase was found in the quiescent period before the event, a sharp drop few days before the earthquake, then a stabilization at a new level until the steady rise begins again. The sharp drop in radon level before the rupture can be judged from microcracking development and the flow of radon in the activation zone that stops with the earthquake and then will relieve the extensional tenses and will stop a further radon decrease. The anomalies recorded present similar characteristics with the ones observed in laboratory experiments on samples under compressive stress^27,29^, however they are of reversed polarity, which may be due to the nature of the extensive field of the study area.

Differences in recording between two neighboring stations, only one of which was installed above a fault that hosts a hydrothermal flow, suggest that deep hydrogeology may play a key role in the measurements. It is possible that the various segments of the fault zone are hydrogeologically connected to each other but also to sites with geothermal manifestations. A station installed near this fault zone’s network can detect stress changes that occur in any part of it.

From the observations made at the stations of the Gulf of Corinth, it became clear that the radon changes that were detected were mostly related to the local fault zones and not distant strong events, even if they were within the radius defined by Dobrovolsky et al.^1^. This in our view is an important conclusion that can assist in the future to follow seismic events in the area and applied to other regions in Greece.

## Methodology

The radon measurements were acquired by 3" × 3" NaI(Tl) scintillation detectors for gamma ray spectrometry. The detectors were positioned at a 1.5 m depth in a shallow cased auger-hole and the acquisition rate was set to 30 min. An automated full spectrum analysis processing is applied to the data for the decomposition of the spectrum to the natural gamma emitting radionuclides in soil—i.e. 40K, daughter nuclides of 222Ra (214Pb, 214Bi) and of 232Th (228Ac, 212Pb, 212Bi and 208Tl)—using the Generalized Least Squares (GLS) method to a matrix of 12 pre-selected energy regions in the energy range of 300 to 2800 keV^56^. All spectrometers were experimentally calibrated (for energy, energy resolution and efficiency) by the manufacturer in water tanks using diluted reference sources of 40K, 226Ra (parent radionuclide of 222Rn) and 232Th, that provide also the corresponding unitary spectra utilized at the spectrometry process. Additional energy auto-calibration was performed for each spectrum prior to the analysis, based on the detection of the dominant photopeak of 40K at 1461 keV, in order to eliminate spectral drifts and minimize deconvolution uncertainties. An alpha radiation spectrometer, consisting on an infiltrated air measurement chamber and an implanted silicon detector with measurement window range 1.5–6 MeV and sensitivity of 55.6 Bq m^−3^ per count hour^−1^, has been used for the inter-calibration of 222Rn activity measured by the gamma radiation monitoring stations to a nominal soil density after their installation. In the gamma-ray measurements the 40K and 232Th concentrations were used as reference of the measurement conditions stability and for radon level correction if necessary. Given that the composition of the soil remained constant during monitoring period, notable alterations in soil water content or soil density, that could affect the detection geometry due to increase of gamma rays self-attention in the ground, would be depicted as a variation of 40K and 232Th activity concentrations. Considering the time-series from all stations, the overall mean relative uncertainty (confidence level of 95%) of each individual measurement was 21%, 16.5% and 31.5% for 40K, 222Rn and 232Th, while when a 12-h moving average filter was drastically reduced to 4.3%, 3.4% and 6.4%, respectively.

At all locations there has been a good coverage by meteorological stations, which provide an accurate monitoring of all parameters that could affect the radon concentration measurements.

In order to correlate the soil radon concentration variation with seismic events, we used the original seismicity catalog of the National Observatory of Athens (NOA), which is the official entity for the seismic monitoring in Greece. The data are freely available through a database of the revised hypocentral solutions in the webpage of NOA: https://bbnet.gein.noa.gr/HL/databases/database.

In the research of precursory phenomena, it is common practice to consider only the seismic events that have the measuring station within their preparation zone^1,57,58^. The Dobrowolski radius^1^ that is usually used in bibliography to determine this zone has mainly derived from earthquake datasets of magnitudes greater than M4.0^1^. Fleiseher^57^ proposed a very similar radius to that of Dobrowolski et al.^1^ for earthquakes larger than M3.0, however, for smaller magnitudes, he proposed another radius much narrower than what could be derived from the Dobrovolsky equation^1^. We selected the events that could cause a measurable anomaly on the radon measurements at LUT station by implementing the criteria of Dobrovolsky et al.^1^ and Fleiseher^57^ (Fig. 4).

To compare the soil radon concentration variations with seismicity, apart from the selected events with the above criteria, we also used the cumulative seismic moment that was calculated from the entire seismicity catalog for the region. The cumulative seismic moment and its daily rate have been also used in the past in bibliography to correlate radon variation with seismicity^22,59^. Because the seismic moment diagrams do not take into account the distance between the earthquakes and the radon station, we indicated this distance with a color scale on the event symbols to assist the interpretation of the correlation diagrams (Fig. 4c).

The seismic events were associated with specific anomalies in the soil radon concentration. These anomalies were characterized by an abrupt drop of the radon level after gradual increase, which were clearly observed in the measurements and are described in detail in the chapter of Results. The anomalies were effectively identified numerically and objectively using an algorithm based on a short-term-average to long-term-average ratio (STA/LTA) taken along the envelope function generated from the radon data time-series. The use of the envelope is usual in such algorithms in seismology^60^. It should be also noted that the upper curve of the envelope function, was little affected by the down-dipping spiky noise in the radon measurements. The STA/LTA has been properly designed to identify the significant anomalies in the long period radon trend.

The abrupt drops of the radon anomalies, which appear as negative slopes in the time series, were identified and isolated by calculating the first-order derivative. The derivative peaks that were higher than the standard deviation were selected and are presented with inverted polarity in Fig. 4c. The onset of these anomalies was detected by calculating the second-order derivative. A cross-correlation was performed a) between the occurrence times of seismic events, which had within their theoretical preparation zone the LUT measuring station and the second-order derivative peaks in order to calculate the lag time between the two data series. The cross-correlation between the daily rate of the cumulative seismic moment and the radon anomalies was also performed providing us with another lag time estimation.
